# Epigenomic regulation of heart failure: integrating histone marks, long noncoding RNAs, and chromatin architecture

**DOI:** 10.12688/f1000research.15797.1

**Published:** 2018-10-29

**Authors:** Timothy A. McKinsey, Thomas M. Vondriska, Yibin Wang

**Affiliations:** 1 Department of Medicine, Division of Cardiology and Consortium for Fibrosis Research & Translation, University of Colorado Anschutz Medical Campus, Aurora, CO, USA; 2 Departments of Anesthesiology, Medicine, and Physiology, David Geffen School of Medicine, University of California, Los Angeles, CA, USA

**Keywords:** epigenetics, cardiac hypertrophy, fibrosis, heart failure

## Abstract

Epigenetic processes are known to have powerful roles in organ development across biology. It has recently been found that some of the chromatin modulatory machinery essential for proper development plays a previously unappreciated role in the pathogenesis of cardiac disease in adults. Investigations using genetic and pharmacologic gain- and loss-of-function approaches have interrogated the function of distinct epigenetic regulators, while the increased deployment of the suite of next-generation sequencing technologies have fundamentally altered our understanding of the genomic targets of these chromatin modifiers. Here, we review recent developments in basic and translational research that have provided tantalizing clues that may be used to unlock the therapeutic potential of the epigenome in heart failure. Additionally, we provide a hypothesis to explain how signal-induced crosstalk between histone tail modifications and long non-coding RNAs triggers chromatin architectural remodeling and culminates in cardiac hypertrophy and fibrosis.

## Introduction

Advances in the pharmacologic management of serum lipid levels and surgical, device-aided, and pharmacologic abatement of myocardial ischemia/reperfusion injury have conspired with increases in obesity to exacerbate the epidemic of heart failure across the developed world. The syndrome of heart failure encompasses a spectrum of symptoms with variable manifestation in different people: impaired ability to meet the oxygen demands of the body are accompanied by impaired contractile function of the heart, myocyte hypertrophy, fibrotic deposition and fibroblast to myofibroblast transformation, and/or metabolic derangements. The scale of the heart failure clinical problem—7 million affected in the United States alone ^1^—combined with the multifarious nature of the pathologic mechanisms make novel molecular mechanisms of cardiac dysfunction a major target for ongoing basic and translational research.

Heart failure is a progressive condition, wherein the cumulative effects of stress to the heart are integrated to alter the function of the organ. While some aspects of cardiac dysfunction can be mitigated by targeting risk factors (e.g. blood pressure control), cellular and molecular insults change the function of the heart in a more lasting and, presently, from a drug development standpoint, intractable manner. To develop the next class of therapeutic targets for heart failure, we need to dissect the influence of pathologic stress on (a) the major classes of cells in the heart and (b) the molecular substrate for persistent cell and organ dysfunction. Towards the latter goal, emerging evidence indicates that chromatin regulatory mechanisms are mobilized following injury to establish and entrench diseased transcriptomes and phenotypes. The families of histone-modifying enzymes that write, read, and erase marks on these proteins in a signal-responsive and locus-specific manner establish the differential accessibility that allows the same genome to encode different cell types. Linking transcription with chromatin regulation, long noncoding RNAs (lncRNAs) have been revealed to exert powerful effects on cardiac cell phenotype through the regulation of gene expression. Together, these and other epigenetic mechanisms impart structure and regulation to chromatin between distinct cell types in the healthy and diseased setting. In this essay, we identify epigenomic regulation of specialized cardiac cell types like myocytes and fibroblasts as promising targets for therapeutic development.

## Targeting histone acetylation for heart failure: HAT, HDAC, and BET inhibitors

Lysine is a versatile amino acid target for post-translational modifications, including methylation, acyl modifications (e.g. acetylation, crotonylation, and succinylation), and small protein isopeptide bonds (e.g. ubiquitination, NEDDylation, and SUMOylation) ^2^. For the purposes of this review, we focus on the acetylation of ε-amino groups of lysine residues within nucleosomal histone tails. This post-translational modification is mediated by histone acetyltransferase (HAT) enzymes and has historically been linked to gene activation, with the charge neutralization provided by acetylation promoting a more permissive environment for transcription by weakening histone:DNA associations and altering histone:histone interactions.

Findings with genetically engineered mice have suggested important roles for the p300 HAT in the control of pathological cardiac remodeling. Heterozygous deletion of p300 was shown to suppress cardiac hypertrophy in response to pressure overload, and overexpression of p300 triggered pathological hypertrophy and heart failure ^3, 4^. Unfortunately, efforts to advance HAT inhibitors as a therapeutic strategy for heart failure have been hampered by the lack of potent and selective pharmacological inhibitors of p300 ^5^. However, a recent virtual screening and medicinal chemistry optimization campaign yielded A-485, an orally bioavailable small molecule inhibitor that is highly selective for p300 and the related HAT, CREB-binding protein (CBP) ^6, 7^. The drug-like properties of A-485 provide an excellent opportunity to assess the efficacy of HAT inhibition in pre-clinical models of pathological cardiac remodeling and thereby determine the translational potential of p300 catalytic activity inhibition for the treatment of heart failure in humans.

A bromodomain, which is an acetyl-lysine binding motif, in p300 is required for chromatin targeting of the HAT ^8, 9^. CBP112 and CBP30 have been developed as small molecules that target the p300 bromodomain and function as acetyl-lysine competitive inhibitors ^10, 11^. An impactful recent study employed proteomics and transcriptomics to quantify acetylation and mRNA and protein abundance in mouse embryonic fibroblasts after cellular p300 inhibition with A-485 versus CBP112 ^12^. Interestingly, gene expression changes triggered by CBP112 were modest compared to those observed upon catalytic inhibition of p300 with A-485, illustrating that the bromodomain of the HAT is required for the regulation of only a subset of target genes. It will be interesting to compare the effects of A-485 and CBP112 in cardiac myocytes to determine the relative contributions of p300 catalytic activity and bromodomain function in the control of pathologic gene expression.

In contrast to HAT inhibitors, a multitude of potent and selective HDAC inhibitors are available ^13^. Most HDAC inhibitors possess a tripartite structure consisting of a zinc-binding group that binds the active site, a linker that mimics the lysine side chain, and a surface recognition cap that confers specificity of the compounds for HDAC enzymes. Distinct genes encode the 18 mammalian HDAC isoforms, which are either zinc dependent (HDACs 1-11) or NAD ^+^ dependent (SirT1–7) ^14^. The original demonstration that HDAC inhibitors could be beneficial in the heart was based on the use of trichostatin A (TSA), a *Streptomyces* metabolite that acts as a pan-inhibitor of zinc-dependent HDACs but does not affect the activity of NAD ^+^-dependent sirtuins ^15– 18^. Subsequently, additional natural products as well as synthetic HDAC inhibitors were shown to be efficacious in animal models of heart failure, blocking pathological cardiac hypertrophy, fibrosis, and inflammation, and improving systolic and diastolic function ^19^.

**Figure 1.**
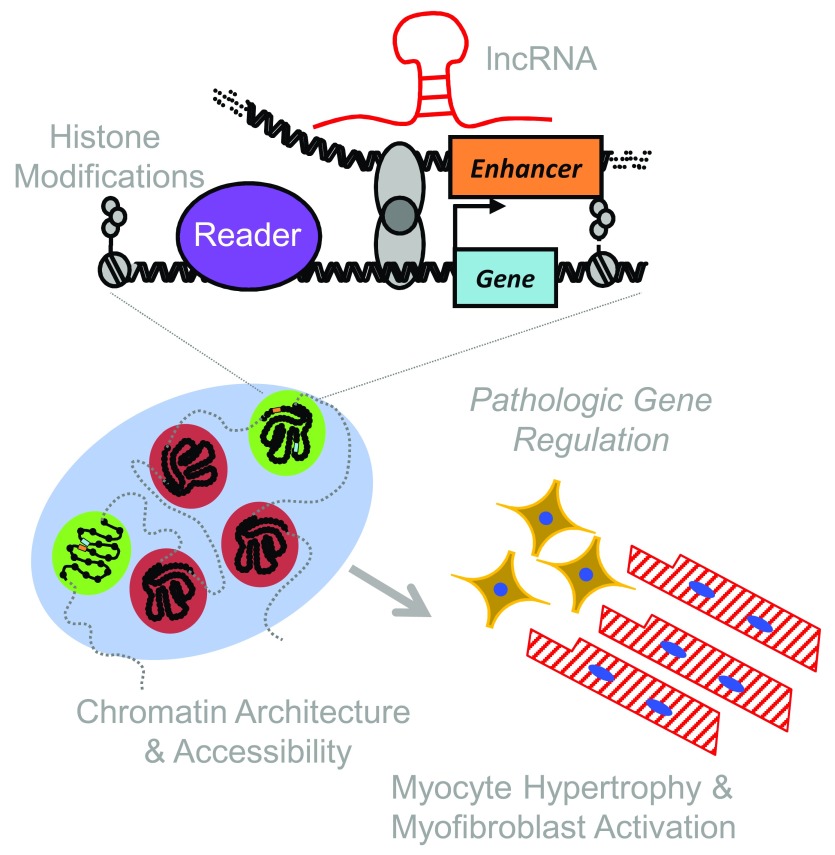
A model for integrating histone marks, long noncoding RNAs (lncRNAs), and chromatin architecture in heart failure. The epigenomic regulation of cardiac phenotype occurs at multiple interacting scales. Histone isoforms, post-translational modification, and nucleosome distribution influence local transcription. lncRNAs have emerged as powerful regulators of gene expression, interacting with chromatin-modifying enzymes and influencing their histone targets. Together with other chromatin regulatory proteins, histone modifications and lncRNAs establish local chromatin accessibility and global chromatin architecture, facilitating short- and long-range regulatory interactions that enable cell type-specific transcriptomes in healthy and diseased conditions.

Remarkably, our understanding of the functions of HDACs in the control of epigenetic regulation of gene expression in heart failure is still extremely limited. A genome-wide evaluation of the impact of HDAC inhibition on one epigenetic mark in normal and stressed hearts was described ^20^. Mice were subjected to left ventricular pressure overload and were administered TSA or vehicle control for four weeks. ChIP-seq of whole heart homogenates with an anti-acetyl-H3K9/K14 antibody revealed that pressure overload broadly altered histone acetylation throughout the genome, and these changes were reversed by TSA. A paradoxical finding from this study was the profound ability of TSA to also reduce H3 acetylation at many loci. These findings suggest the possibility that HDAC activity controls HAT genomic targeting, expression, and/or function in the heart. This mode of crosstalk could explain the seemingly counterintuitive finding that inhibiting enzymes that either add (HAT) or remove (HDAC) acetyl groups can suppress pathogenic processes that contribute to the development of heart failure. Taking this a step further, it is our strong belief that HDACs mediate extensive interplay between diverse epigenetic regulators, including lncRNAs, and coordinate complex remodeling of chromatin architecture in response to pathological stress in cardiac myocytes and fibroblasts, thereby promoting hypertrophy, fibrosis, and ventricular dysfunction ( Figure 1).

Another way to pharmacologically target histone acetylation is through the use of BET protein inhibitors. The most well-characterized family of proteins that ‘read’ acetyl-lysine marks, without also containing catalytic domains for epigenetic modifying activity (e.g. HATs), are the bromodomain and extraterminal domain-containing (BET) proteins (BRD2, BRD3, BRD4, and BRDT). BRD4 and BRDT (testis-specific) harbor a unique carboxy-terminal domain that is able to activate RNA polymerase II (Pol II) by recruiting CDK9, a kinase component of the P-TEFb complex; CDK9 phosphorylates serine-2 of the tail of Pol II, leading to transcription elongation ^21– 23^.

A developing function for BET proteins, in particular BRD4, is the creation of dynamic, cell state-specific enhancers called super-enhancers (SEs). The association of BRD4 with acetyl-H3K27-containing SEs, the signaling of which to proximal promoters is believed to stabilize BRD4-containing coactivator complexes close to transcription start sites, enables P-TEFb-mediated Pol II phosphorylation and transcription elongation. JQ1, which is a small molecule inhibitor that is selective for BET bromodomains, was shown to effectively prevent and reverse cardiac hypertrophy, fibrosis, and ventricular dysfunction, in part, by suppressing the association of BRD4 with SEs associated with pro-hypertrophic and pro-fibrotic genes in the heart ^24– 28^. The extent to which BRD4 genomic targeting in the heart is controlled by distinct HDAC and HAT isoforms has not been determined, nor has the role of BRD4 in coupling to pathogenic lncRNAs and coordinating chromatin architecture remodeling in response to cardiac stress.

The notion of using small molecule inhibitors of epigenetic regulators to treat a chronic condition such as heart failure is often met with doubt, since the regulators to be targeted are widely expressed and mediate fundamental transcriptional mechanisms in many cell types. Nevertheless, there are four FDA-approved HDAC inhibitors, two approved DNMT inhibitors, and several other epigenetic modifying therapies in clinical development for oncologic and non-oncologic indications ^29^. Thus, the feasibility of using ‘epigenetic therapies’ to treat human diseases has been validated, and we believe that this approach has tremendous potential for patients suffering from the complex syndrome of heart failure.

This is also an exciting time to employ chemical biology to elucidate novel epigenetic pathways that control heart failure. No longer are we solely reliant on natural product inhibitors of epigenetic regulators, which often lack selectivity. Exhaustive and sophisticated medicinal chemistry programs in industry and academia have led to the development of highly selective and potent inhibitors of a wide array of epigenetic targets, and many of the compounds are available to the scientific community through programs such as the Structural Genomics Consortium ^30^. Coupling the use of these compounds with well-validated phenotypic assays, such as cell-based assays of cardiomyocyte hypertrophy or fibrosis ^31^, has the potential to rapidly uncover novel roles for epigenetic regulators in the control of heart failure and thus provide crucial mechanistic insights.

## Long non-coding RNAs

lncRNAs are a class of RNA transcripts recently identified to be widely expressed in all tissues. Unlike several functionally and structurally well-defined species of non-coding RNAs, such as rRNA, miRNA, snoRNA, and piRNA, the very definition of lncRNAs remains arbitrary and is often applied to any RNA transcripts that have no coding capacity and are more than 200 nucleotides in length ^32^. Recent progress in transcriptome profiling using next-generation RNA sequencing methods has begun to uncover the enormous scale of the lncRNA products within the cardiac transcriptome and the scope of their contribution to the overall transcriptome reprogramming under physiological and pathological conditions ^33– 37^. In addition to lack of protein coding capacity, common features of cardiac lncRNAs also include low abundance in expression (with notable exceptions) and a lower degree of sequence conservation ^33^. They can be produced from intergenic regions of the genome (intergenic lncRNAs) or from different parts of known genes, such as enhancers, promoters, and exon or intron regions in either sense or anti-sense directions ^38^. It is clear that the transcriptomic complexity of lncRNAs matches, if not surpasses, that of coding mRNAs in the heart.

Because of the scale and complexity of cardiac lncRNAs, physiological or pathological functions for the vast majority of them still remain to be determined. However, recent evidence shows that many cardiac lncRNAs do play important roles in gene regulation during cardiac development and pathophysiology, particularly via epigenomic modulations, as showcased by the prototypic lncRNA H19 in the regulation of Igf2 gene imprinting ^39, 40^.

In the past 5 years, there has been an explosion of new discoveries of epigenomic regulatory lncRNAs, also referred to as epi-lncRNAs, in cardiac development and diseases ^41, 42^. For example, Mhrt and Chaer are reported to regulate chromatin modifications by direct interactions with histone modifiers, such as Brg1 and PRC2 complexes, respectively. The epigenomic impact of such interactions can be either local or more global. For example, Upperhand is a lncRNA that regulates neighboring hand2 gene expression in the developing heart in an allele-specific and cis-regulatory manner ^43^. In contrast, Chaer modulates global histone modifications as an epigenetic check-point for a large number of hypertrophic genes ^44^. Ultimately, the functional outcome of these epi-lncRNAs affects chromatin accessibility for transcription factors, Pol II, and other RNA-processing machinery. In addition to these relatively well-characterized epigenetic modulatory lncRNAs, lncRNAs are also reported to modulate RNA splicing, transportation, and translation ^38^. For example, CHRF and Miat can regulate cardiac gene expression and hypertrophy by binding to and interfering with miRNA functions, serving as so-called miRNA sponges ^45, 46^.

Given the role for lncRNAs in cardiac epigenetic regulation, interfering with lncRNA expression and function can have a significant impact on cardiac pathophysiology. For example, Mhrt expression can block, while Chaer and CHRF inactivation attenuates, pathological cardiac hypertrophy. Manipulation of the expression of the Wisper, Meg3, and MIAT lncRNAs also affects cardiac fibrosis and pathological remodeling ^47– 49^. Tissue and plasma lncRNAs have been identified as potential biomarkers to predict the disease outcome for heart failure ^32, 50– 52^. These studies highlight the therapeutic potential for lncRNA-targeted treatment for heart failure.

LncRNAs are clearly central to the epigenomic network in the heart. However, many challenging issues remain with regard to understanding lncRNA-mediated cardiac epigenetic regulation and translating lncRNA-targeted therapies to the clinic. First, given the fact that the vast majority of lncRNA species have not been functionally annotated, new high-throughout phenotypic screening approaches will be needed to systematically identify functionally important lncRNA species in heart diseases. Second, the mechanisms of lncRNA-mediated epigenetic regulation are extremely diverse and much remains to be discovered. Other than a few exceptions, the structural basis of lncRNA function is still poorly understood ^53, 54^. The detailed molecular processes involved in lncRNA-mediated regulation of histone modifications are largely unknown. In particular, the molecular link between lncRNA function and pathophysiological signaling is still elusive. Finally, many lncRNAs are species specific, leaving concerns about the translational relevance of studies conducted in rodents or other animal models ^55, 56^. Therefore, there are pressing needs to develop novel analytic tools using integrated approaches combined with human datasets to identify and characterize functionally important lncRNAs and to develop novel experimental approaches to characterize lncRNA function in epigenomic regulation by revealing the hidden code of lncRNA structure and identifying the intersections between lncRNAs and epigenomic modulators.

## Chromatin architecture

To get the same genome to behave differently in the presence of identical transcriptional machinery, the hundreds of cell types in mammalian systems must change the interface between these tiers of molecules (i.e. between DNA and everything that regulates it). One manner in which this interface is altered is via structure and accessibility (two separate concepts) of chromatin, in large part through the actions of histone-modifying enzymes as discussed in the first part of this essay. Recent technologies that enable direct measurements of accessibility and structure of the genome reveal how these features are modified in a global, coordinated manner and implicate changes in chromatin architecture as a fundamental driver of disease.

Global changes in histone marks have been observed in multiple studies of animal models of heart failure, including a general trend of increased euchromatic marks and/or decreased heterochromatic marks (early observations from human hearts supported a similar trend ^57^). In mouse models of pressure overload, ChIP-seq has been used to map global deposition of various histone marks associated with transcriptional activation, repression, and enhancer formation in the basal and hypertrophied heart ^58^. Human ^57, 59^ and mouse ^60^ studies of DNA methylation, which is associated with gene expression and may participate in chromatin structure, have also revealed widespread changes during the development of heart failure, with specific localization around genes involved in the disease’s pathogenesis and related to chromatin structural features, such as topologically associated domains ^61^. Chromatin accessibility—that is, the local density of nucleosomes (i.e. how many occupy a fixed region of DNA) and the decoration of nucleosomes with post-translational modifications that facilitate (such as lysine acetylation in particular) or inhibit (such as trimethylation of histone H3 K9 or K27, for instance) transcriptional activity—can be globally assayed by various techniques, which report on the cumulative effects of multiple histone modifications, DNA modifications, and other protein binding rather than being dependent on assumptions based on ChIP-seq data for any single modification. Accessibility is a prerequisite for protein binding and hence transcription. Although it is beyond the scope of this review to comprehensively address this concept, there are scores of histone-modifying enzymes that modulate chromatin in any given cell type while, at the same time, DNA itself is modified, most notably through methylation and hydroxymethylation. These processes do not exist in isolation, and indeed some aspects of DNA methylation can be influenced by chromatin structure, as has been recently reported in cardiac myocytes ^62^. Indeed, the shift at specific loci across the genome between hydroxymethylation and methylation at cytosines has been shown to play a role in pathologic transcriptional changes in cardiac hypertrophy and failure ^63^. Furthermore, heart failure is associated with changes in DNA methylation, which appear to coordinate with chromatin features defined by histone modifications in both mice and humans ^57, 60^.

Chromatin structure, on the other hand, is a three-dimensional problem that incorporates localization within the nucleus (i.e. how close a given region of the genome is to nuclear features such as membranes, nucleoli, or transcription factories), nearness to other regions of the same or different chromosomes (in particular for the actions of transcriptional enhancers, which can act at a distance to regulate transcription during heart failure), and local architectural features that insulate against aberrant transcription while facilitating the appropriate variety. Recent investigations have shown cardiomyocyte chromatin to undergo selective, locus-specific, and highly tuned structural reorganization in the setting of pressure overload hypertrophy in animal models ^64^. Different cardiac cell genes exhibit distinct temporal schemes of chromatin remodeling after pathologic stress ^58, 65^, which have been attributed to underlying chromatin features, such as gene looping, in addition to the actions of enhancer elements. Histone modifications (and the suite of proteins that add, remove, and read these modifications, as well as ATP-dependent chromatin remodelers, which consume ATP to reposition nucleosomes along the genome) and chromatin-binding proteins such as CTCF and high mobility group proteins all contribute to chromatin structure by influencing nucleosome positioning, although a clear rubric for how nucleosome positioning and histone modification directly influence the folding of the genome remains to be determined (the highest resolution studies of chromatin structure across cell types reveal minor differences but reflect that topologically associating domains are largely conserved between lineages ^66^). Indeed, how local chromatin modifications determine local and global epigenomic structure is a fascinating frontier of basic and translational chromatin research. The most compelling evidence for lncRNA-mediated chromatin organization comes from the field of X chromosome inactivation, where the lncRNA *Xist* has been shown to govern inactivation-associated folding of the X chromosome based on its interaction with key sites on the chromosome and the recruitment of inactivated histone modifiers and DNA methylation machinery ^67, 68^.

## Concluding remarks

New classes of therapeutic targets for heart failure require an appreciation of both the multicellular nature of the disease and the distinct pathophysiological mechanisms underpinning the diversity of symptoms in afflicted human populations. As put forth in this essay, we identify the interface among lncRNAs, histone modifications, and chromatin architecture as a key nodal point at which heart failure processes intersect, thus representing a ripe target for novel pharmacologic targeting. Challenges that remain include dissecting the roles of these different epigenetic regulators in different cell types in the heart and determining the optimum chromatin targets for a range of clinical heart failure phenotypes.

## References

[1] Benjamin EJ Blaha MJ Chiuve SE: Heart Disease and Stroke Statistics-2017 Update: A Report From the American Heart Association. *Circulation.* 2017;135(10):e146–e603. 10.1161/CIR.0000000000000485 28122885 PMC5408160

[2] Tan M Luo H Lee S: Identification of 67 histone marks and histone lysine crotonylation as a new type of histone modification. *Cell.* 2011;146(6):1016–28. 10.1016/j.cell.2011.08.008 21925322 PMC3176443

[3] Miyamoto S Kawamura T Morimoto T: Histone acetyltransferase activity of p300 is required for the promotion of left ventricular remodeling after myocardial infarction in adult mice *in vivo*. *Circulation.* 2006;113(5):679–90. 10.1161/CIRCULATIONAHA.105.585182 16461841

[4] Wei JQ Shehadeh LA Mitrani JM: Quantitative control of adaptive cardiac hypertrophy by acetyltransferase p300. *Circulation.* 2008;118(9):934–46. 10.1161/CIRCULATIONAHA.107.760488 18697823 PMC2726266

[5] Wapenaar H Dekker FJ: Histone acetyltransferases: challenges in targeting bi-substrate enzymes. *Clin Epigenetics.* 2016;8:59. 10.1186/s13148-016-0225-2 27231488 PMC4881052

[6] Lasko LM Jakob CG Edalji RP: Discovery of a selective catalytic p300/CBP inhibitor that targets lineage-specific tumours. *Nature.* 2017;550(7674):128–32. 10.1038/nature24028 28953875 PMC6050590

[7] Michaelides MR Kluge A Patane M: Discovery of Spiro Oxazolidinediones as Selective, Orally Bioavailable Inhibitors of p300/CBP Histone Acetyltransferases. *ACS Med Chem Lett.* 2018;9(1):28–33. 10.1021/acsmedchemlett.7b00395 29348807 PMC5767893

[8] Manning ET Ikehara T Ito T: p300 forms a stable, template-committed complex with chromatin: role for the bromodomain. *Mol Cell Biol.* 2001;21(12):3876–87. 10.1128/MCB.21.12.3876-3887.2001 11359896 PMC87051

[9] Ragvin A Valvatne H Erdal S: Nucleosome binding by the bromodomain and PHD finger of the transcriptional cofactor p300. *J Mol Biol.* 2004;337(4):773–88. 10.1016/j.jmb.2004.01.051 15033350

[10] Hammitzsch A Tallant C Fedorov O: CBP30, a selective CBP/p300 bromodomain inhibitor, suppresses human Th17 responses. *Proc Natl Acad Sci U S A.* 2015;112(34):10768–73. 10.1073/pnas.1501956112 26261308 PMC4553799

[11] Picaud S Fedorov O Thanasopoulou A: Generation of a Selective Small Molecule Inhibitor of the CBP/p300 Bromodomain for Leukemia Therapy. *Cancer Res.* 2015;75(23):5106–19. 10.1158/0008-5472.CAN-15-0236 26552700 PMC4948672

[12] Weinert BT Narita T Satpathy S: Time-Resolved Analysis Reveals Rapid Dynamics and Broad Scope of the CBP/p300 Acetylome. *Cell.* 2018;174(1):231–244.e12. 10.1016/j.cell.2018.04.033 29804834 PMC6078418

[13] Roche J Bertrand P: Inside HDACs with more selective HDAC inhibitors. *Eur J Med Chem.* 2016;121:451–83. 10.1016/j.ejmech.2016.05.047 27318122

[14] Gregoretti IV Lee YM Goodson HV: Molecular evolution of the histone deacetylase family: functional implications of phylogenetic analysis. *J Mol Biol.* 2004;338(1):17–31. 10.1016/j.jmb.2004.02.006 15050820

[15] Antos CL McKinsey TA Dreitz M: Dose-dependent blockade to cardiomyocyte hypertrophy by histone deacetylase inhibitors. *J Biol Chem.* 2003;278(31):28930–7. 10.1074/jbc.M303113200 12761226

[16] Bradner JE West N Grachan ML: Chemical phylogenetics of histone deacetylases. *Nat Chem Biol.* 2010;6(3):238–43. 10.1038/nchembio.313 20139990 PMC2822059

[17] Kook H Lepore JJ Gitler AD: Cardiac hypertrophy and histone deacetylase-dependent transcriptional repression mediated by the atypical homeodomain protein Hop. *J Clin Invest.* 2003;112(6):863–71. 10.1172/JCI19137 12975471 PMC193673

[18] Yoshida M Kijima M Akita M: Potent and specific inhibition of mammalian histone deacetylase both *in vivo* and *in vitro* by trichostatin A. *J Biol Chem.* 1990;265(28):17174–9. 2211619

[19] McKinsey TA: Therapeutic potential for HDAC inhibitors in the heart. *Annu Rev Pharmacol Toxicol.* 2012;52:303–19. 10.1146/annurev-pharmtox-010611-134712 21942627

[20] Ooi JY Tuano NK Rafehi H: HDAC inhibition attenuates cardiac hypertrophy by acetylation and deacetylation of target genes. *Epigenetics.* 2015;10(5):418–30. 10.1080/15592294.2015.1024406 25941940 PMC4622459

[21] Brown JD Lin CY Duan Q: NF-κB directs dynamic super enhancer formation in inflammation and atherogenesis. *Mol Cell.* 2014;56(2):219–31. 10.1016/j.molcel.2014.08.024 25263595 PMC4224636

[22] Li G Ruan X Auerbach RK: Extensive promoter-centered chromatin interactions provide a topological basis for transcription regulation. *Cell.* 2012;148(1–2):84–98. 10.1016/j.cell.2011.12.014 22265404 PMC3339270

[23] Zhang Y Wong CH Birnbaum RY: Chromatin connectivity maps reveal dynamic promoter-enhancer long-range associations. *Nature.* 2013;504(7479):306–10. 10.1038/nature12716 24213634 PMC3954713

[24] Anand P Brown JD Lin CY: BET bromodomains mediate transcriptional pause release in heart failure. *Cell.* 2013;154(3):569–82. 10.1016/j.cell.2013.07.013 23911322 PMC4090947

[25] Duan Q McMahon S Anand P: BET bromodomain inhibition suppresses innate inflammatory and profibrotic transcriptional networks in heart failure. *Sci Transl Med.* 2017;9(390): pii: eaah5084. 10.1126/scitranslmed.aah5084 28515341 PMC5544253

[26] Haldar SM McKinsey TA: BET-ting on chromatin-based therapeutics for heart failure. *J Mol Cell Cardiol.* 2014;74:98–102. 10.1016/j.yjmcc.2014.05.002 24838003 PMC4115033

[27] Spiltoir JI Stratton MS Cavasin MA: BET acetyl-lysine binding proteins control pathological cardiac hypertrophy. *J Mol Cell Cardiol.* 2013;63:175–9. 10.1016/j.yjmcc.2013.07.017 23939492 PMC4089995

[28] Stratton MS Lin CY Anand P: Signal-Dependent Recruitment of BRD4 to Cardiomyocyte Super-Enhancers Is Suppressed by a MicroRNA. *Cell Rep.* 2016;16(5):1366–78. 10.1016/j.celrep.2016.06.074 27425608 PMC4972677

[29] Shortt J Ott CJ Johnstone RW: A chemical probe toolbox for dissecting the cancer epigenome. *Nat Rev Cancer.* 2017;17(3):160–83. 10.1038/nrc.2016.148 28228643

[30] Müller S Ackloo S Arrowsmith CH: Donated chemical probes for open science. *eLife.* 2018;7: pii: e34311. 10.7554/eLife.34311 29676732 PMC5910019

[31] Reid BG Stratton MS Bowers S: Discovery of novel small molecule inhibitors of cardiac hypertrophy using high throughput, high content imaging. *J Mol Cell Cardiol.* 2016;97:106–13. 10.1016/j.yjmcc.2016.04.015 27130278 PMC5002372

[32] Bär C Chatterjee S Thum T: Long Noncoding RNAs in Cardiovascular Pathology, Diagnosis, and Therapy. *Circulation.* 2016;134(19):1484–99. 10.1161/CIRCULATIONAHA.116.023686 27821419

[33] Lee JH Gao C Peng G: Analysis of transcriptome complexity through RNA sequencing in normal and failing murine hearts. *Circ Res.* 2011;109(12):1332–41. 10.1161/CIRCRESAHA.111.249433 22034492 PMC3243366

[34] Li D Chen G Yang J: Transcriptome analysis reveals distinct patterns of long noncoding RNAs in heart and plasma of mice with heart failure. *PLoS One.* 2013;8(10):e77938. 10.1371/journal.pone.0077938 24205036 PMC3812140

[35] Ounzain S Micheletti R Beckmann T: Genome-wide profiling of the cardiac transcriptome after myocardial infarction identifies novel heart-specific long non-coding RNAs. *Eur Heart J.* 2015;36(6):353–68a. 10.1093/eurheartj/ehu180 24786300 PMC4320320

[36] He C Hu H Wilson KD: Systematic Characterization of Long Noncoding RNAs Reveals the Contrasting Coordination of *Cis*- and *Trans*-Molecular Regulation in Human Fetal and Adult Hearts. *Circ Cardiovasc Genet.* 2016;9(2):110–8. 10.1161/CIRCGENETICS.115.001264 26896382 PMC4862831

[37] Yang KC Yamada KA Patel AY: Deep RNA sequencing reveals dynamic regulation of myocardial noncoding RNAs in failing human heart and remodeling with mechanical circulatory support. *Circulation.* 2014;129(9):1009–21. 10.1161/CIRCULATIONAHA.113.003863 24429688 PMC3967509

[38] Beermann J Piccoli MT Viereck J: Non-coding RNAs in Development and Disease: Background, Mechanisms, and Therapeutic Approaches. *Physiol Rev.* 2016;96(4):1297–325. 10.1152/physrev.00041.2015 27535639

[39] Pant V Kurukuti S Pugacheva E: Mutation of a single CTCF target site within the *H19* imprinting control region leads to loss of *Igf2* imprinting and complex patterns of de novo methylation upon maternal inheritance. *Mol Cell Biol.* 2004;24(8):3497–504. 10.1128/MCB.24.8.3497-3504.2004 15060168 PMC381662

[40] Liu L An X Li Z: The H19 long noncoding RNA is a novel negative regulator of cardiomyocyte hypertrophy. *Cardiovasc Res.* 2016;111(1):56–65. 10.1093/cvr/cvw078 27084844

[41] Liu J Wang DZ: An epigenetic "LINK(RNA)" to pathological cardiac hypertrophy. *Cell Metab.* 2014;20(4):555–7. 10.1016/j.cmet.2014.09.011 25295782 PMC4886471

[42] Greco CM Condorelli G: Epigenetic modifications and noncoding RNAs in cardiac hypertrophy and failure. *Nat Rev Cardiol.* 2015;12(8):488–97. 10.1038/nrcardio.2015.71 25962978

[43] Anderson KM Anderson DM McAnally JR: Transcription of the non-coding RNA upperhand controls *Hand2* expression and heart development. *Nature.* 2016;539(7629):433–6. 10.1038/nature20128 27783597 PMC5261552

[44] Wang Z Zhang XJ Ji YX: The long noncoding RNA *Chaer* defines an epigenetic checkpoint in cardiac hypertrophy. *Nat Med.* 2016;22(10):1131–9. 10.1038/nm.4179 27618650 PMC5053883

[45] Wang K Liu F Zhou LY: The long noncoding RNA CHRF regulates cardiac hypertrophy by targeting miR-489. *Circ Res.* 2014;114(9):1377–88. 10.1161/CIRCRESAHA.114.302476 24557880

[46] Zhu XH Yuan YX Rao SL: LncRNA MIAT enhances cardiac hypertrophy partly through sponging miR-150. *Eur Rev Med Pharmacol Sci.* 2016;20(17):3653–60. 27649667

[47] Micheletti R Plaisance I Abraham BJ: The long noncoding RNA *Wisper* controls cardiac fibrosis and remodeling. *Sci Transl Med.* 2017;9(395): pii: eaai9118. 10.1126/scitranslmed.aai9118 28637928 PMC5643582

[48] Piccoli MT Gupta SK Viereck J: Inhibition of the Cardiac Fibroblast-Enriched lncRNA *Meg3* Prevents Cardiac Fibrosis and Diastolic Dysfunction. *Circ Res.* 2017;121(5):575–83. 10.1161/CIRCRESAHA.117.310624 28630135

[49] Qu X Du Y Shu Y: MIAT Is a Pro-fibrotic Long Non-coding RNA Governing Cardiac Fibrosis in Post-infarct Myocardium. *Sci Rep.* 2017;7: 42657. 10.1038/srep42657 28198439 PMC5309829

[50] Kumarswamy R Bauters C Volkmann I: Circulating long noncoding RNA, LIPCAR, predicts survival in patients with heart failure. *Circ Res.* 2014;114(10):1569–75. 10.1161/CIRCRESAHA.114.303915 24663402

[51] de Gonzalo-Calvo D Kenneweg F Bang C: Circulating long-non coding RNAs as biomarkers of left ventricular diastolic function and remodelling in patients with well-controlled type 2 diabetes. *Sci Rep.* 2016;6: 37354. 10.1038/srep37354 27874027 PMC5118808

[52] Fan Z Gao S Chen Y: Integrative analysis of competing endogenous RNA networks reveals the functional lncRNAs in heart failure. *J Cell Mol Med.* 2018;22(10):4818–4829. 10.1111/jcmm.13739 30019841 PMC6156393

[53] Yan K Arfat Y Li D: Structure Prediction: New Insights into Decrypting Long Noncoding RNAs. *Int J Mol Sci.* 2016;17(1): pii: E132. 10.3390/ijms17010132 26805815 PMC4730372

[54] Sallam T Sandhu J Tontonoz P: Long Noncoding RNA Discovery in Cardiovascular Disease: Decoding Form to Function. *Circ Res.* 2018;122(1):155–66. 10.1161/CIRCRESAHA.117.311802 29301847 PMC5902384

[55] Freedman JE Miano JM, National Heart, Lung, and Blood Institute Workshop Participants*: Challenges and Opportunities in Linking Long Noncoding RNAs to Cardiovascular, Lung, and Blood Diseases. *Arterioscler Thromb Vasc Biol.* 2017;37(1):21–5. 10.1161/ATVBAHA.116.308513 27856459 PMC5222717

[56] Lucas T Bonauer A Dimmeler S: RNA Therapeutics in Cardiovascular Disease. *Circ Res.* 2018;123(2):205–20. 10.1161/CIRCRESAHA.117.311311 29976688

[57] Movassagh M Choy MK Knowles DA: Distinct epigenomic features in end-stage failing human hearts. *Circulation.* 2011;124(22):2411–22. 10.1161/CIRCULATIONAHA.111.040071 22025602 PMC3634158

[58] Papait R Cattaneo P Kunderfranco P: Genome-wide analysis of histone marks identifying an epigenetic signature of promoters and enhancers underlying cardiac hypertrophy. *Proc Natl Acad Sci U S A.* 2013;110(50):20164–9. 10.1073/pnas.1315155110 24284169 PMC3864351

[59] Meder B Haas J Sedaghat-Hamedani F: Epigenome-Wide Association Study Identifies Cardiac Gene Patterning and a Novel Class of Biomarkers for Heart Failure. *Circulation.* 2017;136(16):1528–44. 10.1161/CIRCULATIONAHA.117.027355 28838933

[60] Chen H Orozco LD Wang J: DNA Methylation Indicates Susceptibility to Isoproterenol-Induced Cardiac Pathology and Is Associated With Chromatin States. *Circ Res.* 2016;118(5):786–97. 10.1161/CIRCRESAHA.115.305298 26838786 PMC4779427

[61] Gilsbach R Schwaderer M Preissl S: Distinct epigenetic programs regulate cardiac myocyte development and disease in the human heart *in vivo*. *Nat Commun.* 2018;9(1): 391. 10.1038/s41467-017-02762-z 29374152 PMC5786002

[62] Nothjunge S Nührenberg TG Grüning BA: DNA methylation signatures follow preformed chromatin compartments in cardiac myocytes. *Nat Commun.* 2017;8(1): 1667. 10.1038/s41467-017-01724-9 29162810 PMC5698409

[63] Greco CM Kunderfranco P Rubino M: DNA hydroxymethylation controls cardiomyocyte gene expression in development and hypertrophy. *Nat Commun.* 2016;7: 12418. 10.1038/ncomms12418 27489048 PMC4976219

[64] Rosa-Garrido M Chapski DJ Schmitt AD: High-Resolution Mapping of Chromatin Conformation in Cardiac Myocytes Reveals Structural Remodeling of the Epigenome in Heart Failure. *Circulation.* 2017;136(17):1613–25. 10.1161/CIRCULATIONAHA.117.029430 28802249 PMC5648689

[65] Sayed D He M Yang Z: Transcriptional regulation patterns revealed by high resolution chromatin immunoprecipitation during cardiac hypertrophy. *J Biol Chem.* 2013;288(4):2546–58. 10.1074/jbc.M112.429449 23229551 PMC3554922

[66] Schmitt AD Hu M Jung I: A Compendium of Chromatin Contact Maps Reveals Spatially Active Regions in the Human Genome. *Cell Rep.* 2016;17(8):2042–59. 10.1016/j.celrep.2016.10.061 27851967 PMC5478386

[67] Lee JT: Epigenetic regulation by long noncoding RNAs. *Science.* 2012;338(6113):1435–9. 10.1126/science.1231776 23239728

[68] Engreitz JM Pandya-Jones A McDonel P: The Xist lncRNA exploits three-dimensional genome architecture to spread across the X chromosome. *Science.* 2013;341(6147):1237973. 10.1126/science.1237973 23828888 PMC3778663

